# Mesoporous-Layered Double Oxide/MCM-41 Composite with Enhanced Catalytic Performance for Cyclopentanone Aldol Condensation

**DOI:** 10.3390/molecules28237920

**Published:** 2023-12-03

**Authors:** Jinfan Yang, Ning Shang, Jiachen Wang, Huimin Liu

**Affiliations:** College of Bioresources Chemical and Materials Engineering, Shaanxi University of Science & Technology, Xi’an 710021, China; 220112111@sust.edu.cn (N.S.); 230112130@sust.edu.cn (J.W.); 230112116@sust.edu.cn (H.L.)

**Keywords:** cyclopentanone, aldol condensation, hydrotalcite, MCM-41

## Abstract

Layered double oxides are widely employed in catalyzing the aldol condensation for producing biofuels, but its selectivity and stability need to be further improved. Herein, a novel MCM-41-supported Mg–Al-layered double oxide (LDO/MCM-41) was prepared via the in situ integration of a sol–gel process and coprecipitation, followed by calcination. This composite was first employed to catalyze the self-condensation of cyclopentanone for producing high-density cycloalkane precursors. LDO/MCM-41 possessed large specific surface area, uniform pore size distribution, abundant medium basic sites and Bronsted acid sites. Compared with the bulk LDO, LDO/MCM-41 exhibited a higher selectivity for C10 and C15 oxygenates at 150 °C (93.4% vs. 84.6%). The selectivity for C15 was especially enhanced on LDO/MCM-41, which was three times greater than that on LDO. The stability test showed that naked LDO with stronger basic strength had a rapid initial activity, while it suffered an obvious deactivation due to its poor carbon balance. LDO/MCM-41 with lower basic strength had an enhanced stability even with a lower initial activity. Under the optimum conditions (50% LDO loading, 170 °C, 7 h), the cyclopentanone conversion on LDO/MCM-41 reached 77.8%, with a 60% yield of C10 and 15.2% yield of C15.

## 1. Introduction

The increasing concerns about the environmental and energy issues has promoted the valorization of renewable biomass to value-added biofuels and chemicals [1,2,3]. Lignocellulose, the main component of agricultural waste and forest residues, is one of the most potential biomasses due to its abundant source and cheap price [3]. Most lignocellulose-derived platform chemicals (e.g., furfural, acetone, levulinic acid) are small molecules of less than six carbon atoms; therefore, carbon–carbon coupling reaction is essential to upgrade them into larger molecules for various applications [4,5,6,7]. In this field, great efforts have been made by Dumesic [8,9], who first transformed furfural and other platform chemicals into diesel range alkanes via aldol condensation, followed by hydrogenation. These alkanes prepared from furfural were mostly chain alkanes with low density (<0.77 g mL^−1^); therefore, the synthesis of renewable high-density cyclic alkanes is attracting more attention for satisfying the specification of jet fuels [6,7,10,11,12].

Cyclopentanone (CPO) is an important platform chemical obtained from the selective hydrogenation of furfural. It possesses a ring structure and two carbon atoms with α-H, making it a promising building block for producing cyclic alkanes [11,12]. To date, various heterogeneous metal oxide catalysts (e.g., MgO, CaO, CeO_2_, ZrO_2_, TiO_2_-ZrO_2_, MgO-ZrO_2_) have been developed to catalyze the aldol condensation of CPO for producing cyclic fuel precursors [10,13,14,15]. The acid-base properties of catalyst have great impact on its activity and selectivity. In this regard, our group once found that Mg–Al-layered double oxides (LDOs) had a higher catalytic activity than the alkaline earth oxides in the self-condensation of CPO, owing to its acid-base synergy effect. The obtained dimer (C10 oxygenate) further converted into high-density cycloalkanes (0.86 g mL^−1^) after hydrodeoxygenation [7]. LDO is the calcined product of an anionic clay called layered double hydroxides (LDHs), whose general formula is [M(ii)_1−x_M(iii)_x_(OH)_2_]^x+^(A^n−^_x/n_)·mH_2_O [16]. LDO materials have adjustable element compositions and acid-base properties, causing them to be widely employed in catalyzing various organic reactions [17]. However, it is worth noting that in the LDO-catalyzed CPO condensation reaction, some large oligomers were generated and accumulated on the catalyst, leading to a low selectivity and catalyst stability. Furthermore, the trimer (C15 oxygenate) product was hardly obtained in the reaction, though a trimer was more valuable than a dimer as a high-density jet fuel precursor.

The modification of the surface physicochemical microenvironment of a catalyst is an effective method to optimize its catalytic performance. In this respect, introducing mesoporous structure into catalysts could create new active sites and facilitate the diffusion of large compounds [15,18,19]. For example, Faba et al. [18] found that high-surface-area mesoporous graphite-supported Mg–Zr oxides showed higher activity and selectivity than the bulk Mg–Zr oxides in the furfural–acetone aldol condensation. The improved performance was attributed to the more favorable basic site distribution and the greater interaction of the reactants with the carbon surface. The deactivation which resulted from the coke deposition was greatly inhibited by the tuning of the carbon support’s morphology. Besides the carbon support, mesoporous silicas (e.g., SBA-15, MCM-41) are widely used as heterogeneous catalyst supports owing to their large surface area, ordered porous channels with uniform pore size distribution, and relatively high thermal stability [20]. It was reported that the dispersion of metal oxides on the inert silica could create the structural defects, leading to the presence of new acid or basic sites. Furthermore, the mesoporous character of silicas could facilitate the homogeneous dispersion of mixed oxides and the diffusion of large molecules [18]. According to the literature, the mesoporous silica-supported LDH/LDO composites have been utilized in the aliphatic ester synthesis [21,22], chromene synthesis [23], CO_2_ capture [16,24], and pyrolysis [25].

To the best of our knowledge, the effect of mesoporous silica on the catalytic performance of LDOs was never studied in the self-condensation of CPO. The main aim of the present study was to improve the catalytic performance of LDO materials with a unique strategy. Herein, a novel MCM-41-supported Mg–Al-layered double oxide composite (LDO/MCM-41) was successfully prepared via an in situ integration of a sol–gel process and coprecipitation, followed by calcination. The resulting mesoporous LDO/MCM-41 was first studied in the CPO self-condensation, and the intrinsic reasons for its excellent catalytic performance were discussed based on detailed characterizations. The effects of reaction time, reaction temperature, and LDO loading were also investigated.

## 2. Results and Discussions

### 2.1. XRD

**Figure 1:**
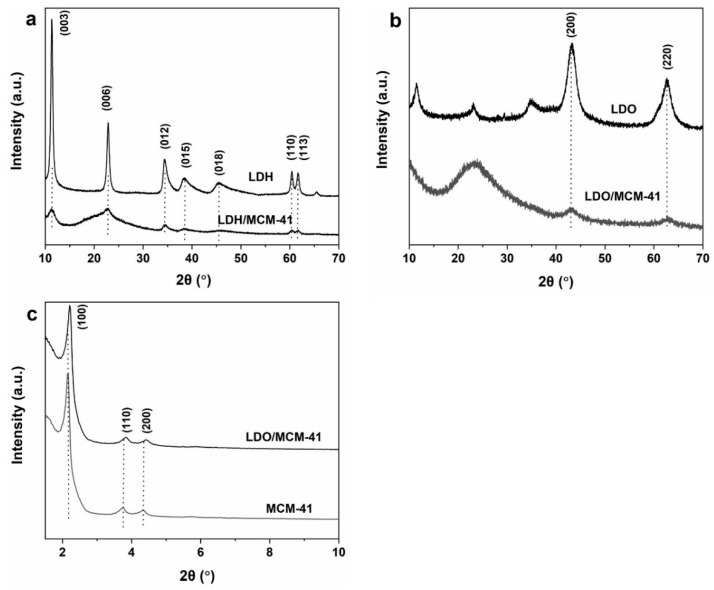
(**a**) XRD patterns of samples before calcination. (**b**) XRD patterns of LDO and LDO/MCM-41. (**c**) Low-angle XRD patterns of MCM-41 and LDO/MCM-41.

The successful incorporation of LDH into silica matrix was confirmed by the XRD patterns of LDH and LDH/MCM-41. As shown in Figure 1a, the presence of three intense reflection peaks at 2θ values of 11.5°, 23.43°, and 34.3° were ascribed to the (003), (006), and (012) planes of a typical hydrotalcite-like structure [26]. The weaker and wider peaks of LDH/MCM-41 demonstrates that the incorporation of silica source in the coprecipitation process affected the crystallization of hydrotalcite, leading to a smaller crystalline size of hydrotalcite. Figure 1b shows that the hydrotalcite-like phase disappeared after calcination and LDH/MCM-41 was transformed into LDO/MCM-41. A broad peak at 22.6° on LDO/MCM-41 corresponded to the amorphous silica [27]. Meanwhile, two peaks at 42.1° and 62.1°on LDO/MCM were assigned to the (2 0 0) and (2 2 0) planes of MgAlO phase (JCPDS 43-1022), formed by the dissolution of Al^3+^ [26]. The crystal size of LDO/MCM-41 and LDO was 3.0 and 10.5 nm, respectively, using the Scherrer equation.

Furthermore, low-angle XRD patterns in Figure 1c shows that both MCM-41 and LDO/MCM-41 had a typical hexagonal lattice structure of mesopores as evident from three narrow reflections at 2θ = 2.3° (100), 3.7° (110), and 4.3° (200). The lower intensity of LDO/MCM-41 can be explained by its lower degree of pore ordering and change in the scattering contrast in the metal-incorporated MCM-41 samples [20].

### 2.2. N_2_ Sorption

**Figure 2:**
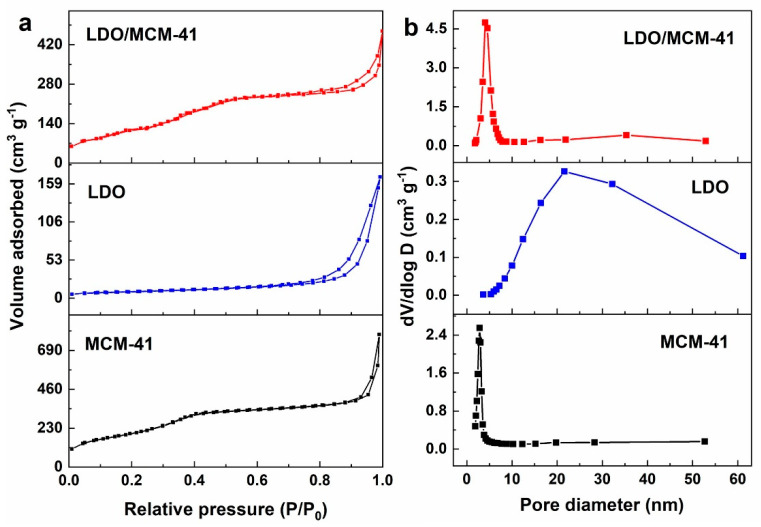
(**a**) Nitrogen adsorption−desorption isotherms and (**b**) pore size distribution curve of different samples.

The N_2_ adsorption–desorption isotherms of the samples are shown in Figure 2a. According to the IUPAC classification [28], MCM-41 sample had the hybrid features of type IVa and type IVb isotherms, and LDO/MCM-41 sample was more like a type IVa isotherm. It is reported that type IVa isotherm accompanied by hysteresis occurs when the pore width exceeds 4 nm, and a completely reversible type IVb isotherm occurs with adsorbents having mesopores of smaller width. Both MCM-41 and LDO/MCM-41 displayed H1 hysteresis loop, being characterized by cylindrical homogeneous mesoporous material with a narrow pore size distribution. On the other hand, LDO sample had a typical type IVa isotherm with H3 hysteresis loop, being characterized by irregular pores with a slit shape. Loops of this type are given by the non-rigid aggregates of plate-like particles (e.g., hydrotalcites). According to the quantitative data summarized in Table 1, the BET surface area and pore volume of LDO/MCM-41 were determined to be 541 m^2^ g^−1^ and 0.50 cm^3^ g^−1^, respectively, which were larger than those of LDO (150 m^2^ g^−1^ and 0.18 cm^3^ g^−1^) but smaller than those of MCM-41 (738 m^2^ g^−1^ and 0.65 cm^3^ g^−1^). This result proves the successful incorporation of LDO nanoparticles on the MCM-41 support. The pore size distribution evaluated via the BJH model is shown in Figure 2b. It is found that LDO had a wide pore size distribution from 2 to 60 nm. Instead, the pore size distribution of LDO/MCM-41 was significantly reduced to 2~9 nm. The average pore size of LDO/MCM-41, LDO, and MCM-41 were determined to be 6.1 nm, 15.2 nm, and 3.5 nm, respectively. This implies that for LDO/MCM-41 composite, most of the mesopores corresponding to the hydrotalcite disappeared while those corresponding to the MCM-41 were somewhat modified.

### 2.3. SEM

**Figure 3:**
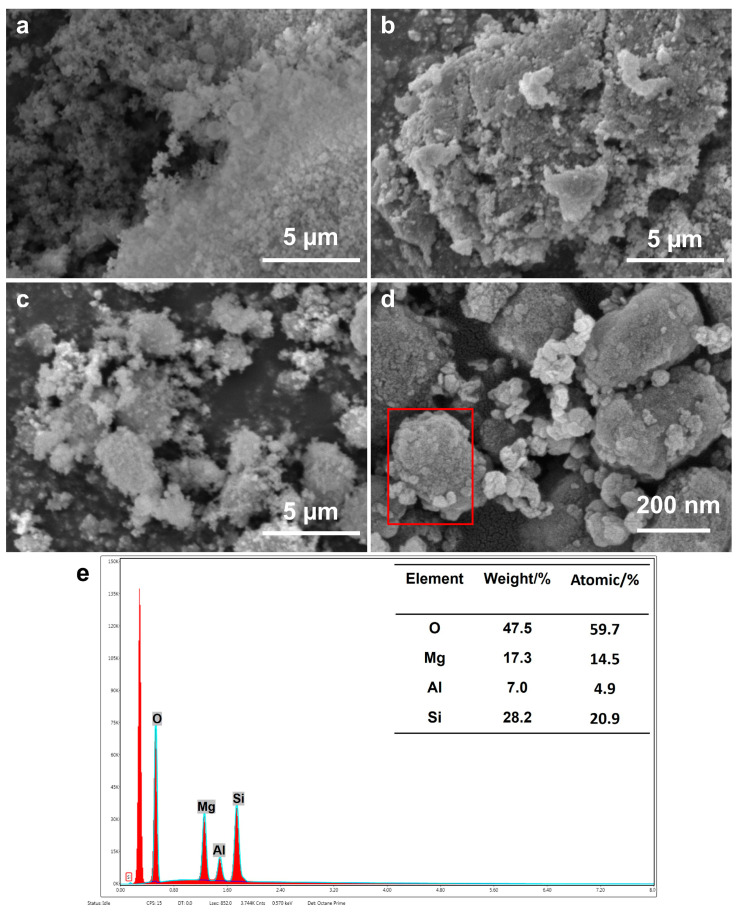
SEM images of (**a**) MCM-41, (**b**) LDO, (**c**,**d**) LDO/MCM-41, and (**e**) SEM–EDS analysis of the specified red box area.

The morphology of LDO/MCM-41 and its control samples were examined via SEM. As shown in Figure 3a,b, the naked MCM-41 contained uniform spherical particles, forming a sponge-like porous nature. The naked LDO presented rough agglomerates composed of irregular nanoparticles, which probably impeded the mass transfer in the condensation involving large-molecular compounds. Based on the SEM images of LDO/MCM-41 shown in Figure 3c,d, MgAl-LDO, characterized by small flake-shaped particles, dispersed well on the surface of the mesoporous silica that is composed by crystals of smooth surfaces and rounded edges (red box). Furthermore, a small number of isolated LDO agglomerates was also observed. SEM–EDS analysis in Figure 3e shows that the elements Mg, Al, and Si were simultaneously detected in the specified area, indicating the successful incorporation of metallic active sites on the silicas support. The SEM–EDS mapping of LDO/MCM-41 (Figure S1) further confirms the thorough dispersion of MgAl oxides on MCM-41, which was in accordance with the XRD results. Thus, this in situ preparation method effectively influenced the aggregation morphology of the composite.

### 2.4. TPD and Pyridine-FTIR

**Figure 4:**
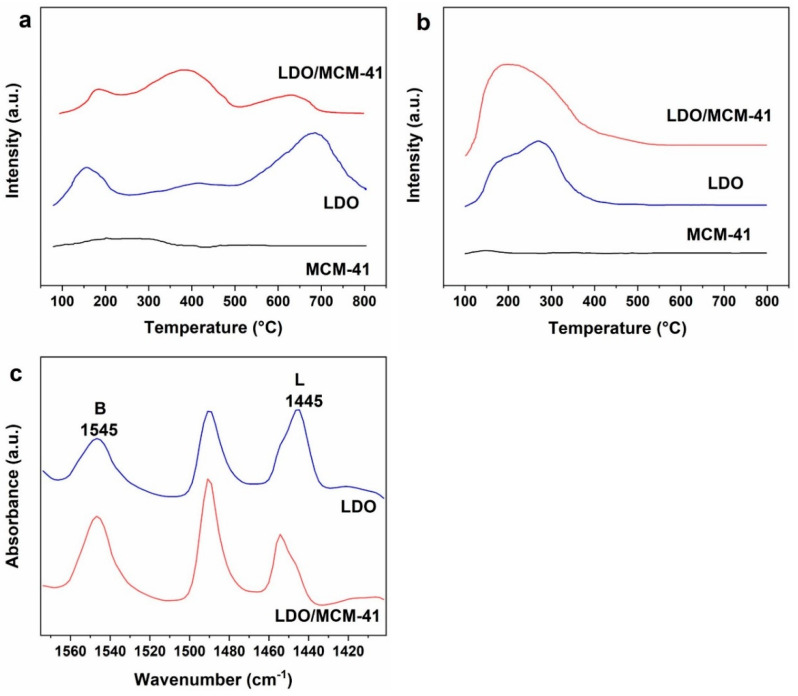
(**a**) CO_2_-TPD, (**b**) NH_3_-TPD, (**c**) Py-FTIR of different samples.

CO_2_-TPD was performed to probe the basic site distribution of LDO/MCM-41 and its control samples. The volume of desorbed CO_2_ with increasing temperature represented the basic sites’ amounts with different strengths. As shown in Figure 4a, the desorption peak on MCM-41 was negligible, owing to its deficiency of active sites. The profile of LDO and LDO/MCM-41 could be divided into three types of basic sites: weak (150 °C), medium (360 °C), and strong (>600 °C) [29]. The weak basic sites were associated with the surface hydroxyl groups. The medium and strong sites were associated with M^2+^–O^2–^ pairs and isolated O^2−^ anions [6]. Compared with LDO, LDO/MCM-41 had a noticeably stronger peak corresponding to medium basic sites, and the peak corresponding to strong basic sites located at lower temperature.

Based on the quantitative analysis of basic sites summarized in Table 2, the total amount of basic sites on LDO was 0.62 mmol g^−1^ and the strong basic sites were dominant (0.36 mmol g^−1^). In contrast, LDO/MCM-41 had a lower basic site amount of 0.43 mmol g^−1^ and the medium basic sites dominated on the catalyst surface (0.25 mmol g^−1^). The result indicates that the LDO/MCM-41 had a weaker strength of basic sites than LDO, and the incorporation of MCM-41 increased the proportion of medium basic sites on the catalyst surface. The decreased strong basic sites on LDO/MCM-41 could be explained by the well-dispersed mixed oxides in the siliceous framework of MCM-41 [5]. Shao once reported that the formation of CPO condensation products depended on the weak and medium basic sites, instead of strong basic sites on catalysts [30]. Thus, the increased medium-strength basic sites were expected to be advantageous for the CPO condensation.

It is known that the basic sites were involved in the CPO activation and the acid sites contributed to the selectivity toward condensation products [13,31]. Thus, NH_3_-TPD was employed to probe the acid properties of catalysts. Figure 4b shows that both LDO and LDO/MCM-41 displayed a broad desorption signal below 350 °C, which corresponded to the weak medium acid sites. Notably, the total amount of acid sites on LDO/MCM-41 was higher than LDO (0.77 vs. 0.60 mmol g^−1^, Table 2). Furthermore, the nature of the acid sites on LDO and LDO/MCM-41 was determined via pyridine-FTIR. As shown in Figure 4c, the bands at 1545 cm^−1^ and 1445 cm^−1^ were attributed to coordination of pyridine molecule with Bronsted and Lewis acidic sites, respectively [32]. It is observed that both LDO and LDO/MCM-41 possessed Bronsted and Lewis acid sites. More importantly, the ratios of Bronsted to Lewis acidic sites (n_B_/n_L_) increased from 1.40 on LDO to 2.37 on LDO/MCM-41. The increased Bronsted acid sites on LDO/MCM-41 could be explained by the isomorphous substitution of Si with Al and Mg producing negatively charged frameworks. The Bronsted acidity was known to appear upon the presence of an excess of the negative charge [33]. Therefore, the incorporation of MCM-41 into LDO influenced the number and nature of acid sites on the catalyst surface.

### 2.5. Activity Evaluation

**Figure 5:**
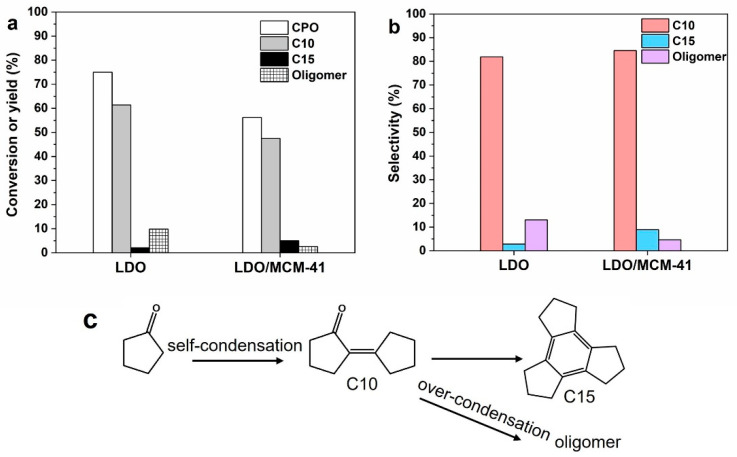
(**a**) Comparison of conversion (white bar), yield of C10 yield (grey bar), C15 (black bar), and oligomer (patterned bar) on LDO and LDO/MCM-41. (**b**) Selectivity of C10 (red bar), C15 (blue bar), and oligomer (purple bar). Reaction conditions: 0.2 g catalyst, 2.0 g CPO, 160 °C, 5 h. (**c**) The reaction route of CPO self- condensation.

The catalytic behaviors of LDO/MCM-41 and the control samples were investigated in the self-condensation of CPO. After reacting for 5 h at 160 °C, no CPO was consumed on MCM-41 due to its neutral property. Figure 5a shows that the conversion on LDO reached 75.0%, resulting in a C10 yield of 61.4% and a C15 yield of 2.1%. When the equal mass of LDO/MCM-41 was employed, the CPO conversion decreased to 56.2% due to the less active sites of the composite. The yields of C10 and C15 oxygenates were 47.5% and 5.0%, respectively. Figure 5b shows that the selectivity of target products was obviously increased from 84.6% on LDO to 93.4% on LDO/MCM-41, and the selectivity of oligomers decreased from 12.9% on LDO to 4.6% on LDO/MCM-41. In particular, LDO/MCM-41 had a C15 selectivity three times greater than LDO. The result indicates that the over-condensation of CPO was successfully inhibited on LDO/MCM-41, leading to an improved atom economy.

Based on the previous characterizations, the optimized physicochemical environment on LDO/MCM-41 endowed its outstanding selectivity. Firstly, a large amount of medium-strength basic sites on LDO/MCM facilitated the controllable condensation of CPO, as medium basic sites were known to be the active sites for this reaction [30,34]. In the aldol condensation of CPO, the basic O sites should be strong enough to abstract α-H atom of the adsorbed CPO molecule to form an enolate species, which would react with another CPO molecule in the following nucleophilic addition. However, basic sites that are too strong would induce the easy deprotonation of unsaturated dimeric or trimeric ketone products, leading to the accumulation of oligomers on the active sites [15,35]. For instance, CPO was completely consumed by aqueous NaOH, but its selectivity to C10 was only 19.7% [36]. Accordingly, the poor selectivity of LDO was rationalized by its large amount of strong basic sites on catalyst (confirmed by TPD). Secondly, it is known that acid sites could help stabilize the enolate intermediate and facilitate the H abstraction through basic sites. The larger amount of acid sites on LDO/MCM-41 especially Bronsted acid would stabilize the CPO molecule via hydrogen bonding, thus facilitating the C–C coupling between the enol intermediates and another CPO molecule. It is found that large oligomers tended to accumulate at Lewis acid sites [37].

### 2.6. Stability

As the oligomers byproduct would deposit on the active sites and deactivate the catalyst, the stability of LDO/MCM-41 was investigated according to the method described by Resasco [14,15]. Herein, the catalyst stability in a batch reactor was evaluated by the evolution of conversion with the product of catalyst dosage and reaction time (m*t) for two different catalyst dosages. When the catalyst was stable, the conversions with two different catalyst dosages (m_1_ and m_2_) were the same after reacting for t_1_ and t_2_ if m_1_*t_1_ = m_2_*t_2_. However, when the catalyst deactivated with time, the conversions at a given m*t were different. That is, a lower catalyst dosage required a longer time to reach a certain conversion, thus the catalyst would exhibit more obvious deactivation than that with a higher catalyst dosage and a shorter reaction time. Based on this method, a 0.05 g catalyst was used for 4 h in the CPO condensation and compared with a 0.1 g catalyst that was reacted for 2 h. As shown in Table 3, the conversions on two different LDO dosages were quite different while the conversions on LDO/MCM-41 were almost the same, indicating an obvious deactivation on LDO and a good stability on LDO/MCM-41. Moreover, LDO/MCM-41 always kept a better carbon balance than LDO at different reaction conditions. The calculated turnover frequency (TOF) based on the initial reaction rate shows that LDO had a higher TOF than LDO/MCM-41, which was probably the intrinsic reason for the lower stability of LDO, because the large amount of strong basic sites on LDO not only improved reaction rate, but also the formation and accumulation of over-condensed products [38].

### 2.7. Effect of Reaction Parameters

**Figure 6:**
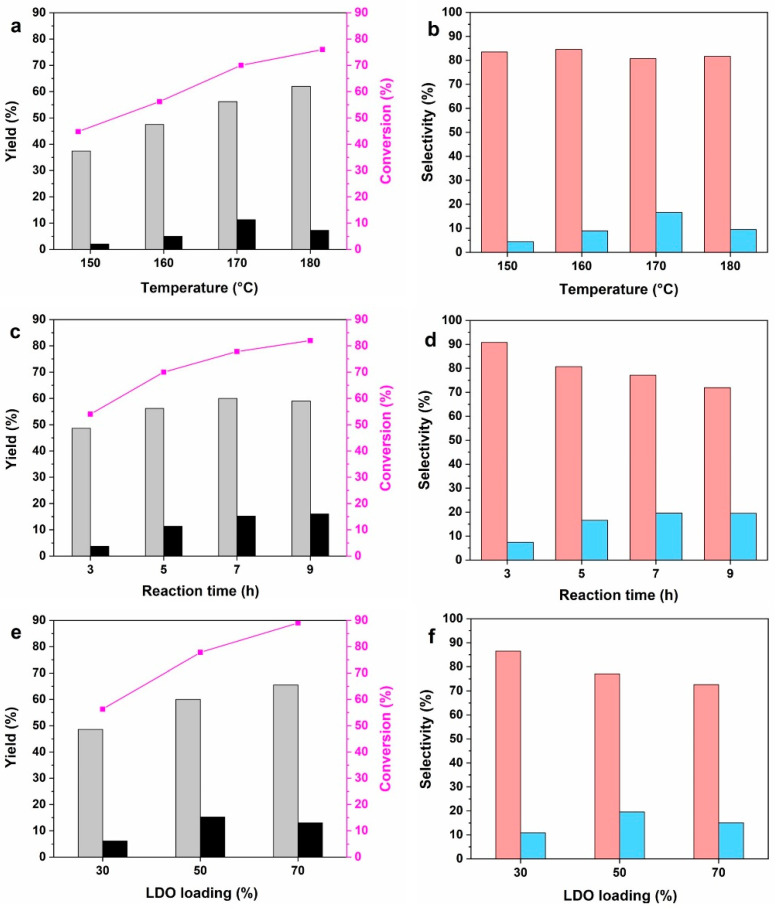
(**a**,**b**) Effect of reaction temperature. Reaction conditions: 5 h, 1.0 g CPO, 0.1 g LDO/MCM-41. (**c**,**d**) Effect of reaction time. Reaction conditions: 170 °C, 1.0 g CPO, 0.1 g LDO/MCM-41. (**e**,**f**) Effect of LDO loading. Reaction conditions: 170 °C, 7 h, 1.0 g CPO, 0.1 g LDO/MCM-41. Yield of C10 (grey bar) and C15 (black bar), selectivity of C10 (red bar) and C15 (blue bar).

The reaction temperature had a great impact on the LDO/MCM-41-catalyzed self-condensation of CPO. As shown in Figure 6a, when the reaction temperature was 150 °C, the conversion on LDO/MCM-41 was only 44.8% and the total yield of target products was 39.4% (37.4% C10, 2.0% C15). Increasing the temperature from 150 °C to 170 °C greatly facilitated the aldol condensation as it was an endothermic reaction process. At 170 °C, the conversion increased to 70.0% and the yield of target products reached the maximum of 67.2% (56.2% C10, 11.0% C15). When the temperature further increased to 180 °C, the conversion increased while the total yield of C10 and C15 remained stable, owing to the formation of over-condensed oligomers at a harsh reaction condition [13,36]. The selectivity of C10 and C15 as a function of reaction temperature was shown in Figure 6b. It is observed that the C10 selectivity basically kept stable at 80% and the C15 selectivity improved clearly with the increasing temperature, reaching the maximum at 170 °C.

As the formation of C15 oxygenate was a cascade reaction, the effect of reaction time on the catalytic performance was also investigated. Figure 6c shows that when the time was prolonged from 3 to 7 h, the conversion increased from 54.0% to 77.8%, and the total yield of products increased from 52.3% to 75.2%. The positive effect on conversion and yield was weakened with a further increase in the reaction time to 9 h. Figure 6d further proves that the C10 selectivity decreased and the C15 selectivity increased with time, reaching the optimal ratio at 7 h. Additionally, the catalytic performance of LDO was compared under the optimal reaction conditions (170 °C, 7 h). It is found that CPO was completely consumed on LDO, presenting an 82.0% yield of C10 and a 2.5% yield of C15. The total selectivity of target products on LDO was 84.5%, which was approximately 12.0% lower than that on LDO/MCM-41.

The effect of LDO loading on the catalytic performance of LDO/MCM-41 composites was investigated under the optimal reaction conditions. Figure 6e shows that with the increased LDO loading in the composite, the conversion of CPOon 30LDO, 50LDO, and 70LDO were 56.3%, 77.8% and 89.0%, respectively. The total yield of C10 and C15 also had a growing trend, and it reached a plateau at 70LDO. Figure 6f reveals that both 30LDO and 50LDO had an excellent total selectivity over 96.0%; however, the selectivity on 70LDO was only 88.2%. Accordingly, it is concluded that the catalytic performance of LDO/MCM-41 highly depended on the LDO loading. The phenomenon was rationalized by the combined effect of the chemical property and physical structure of LDO/MCM-41 composite. First, based on the quantitative analysis of CO_2_-TPD (Table S1), the total basic sites’ amount of different LDO/MCM-41 composites, especially strong basic sites, significantly increased with increasing LDO loading (70LDO, 0.27 mmol > 50LDO, 0.12 mmol > 30LDO, 0.05 mmol). The increased strong basic sites not only facilitated the activation of CPO molecules, but also strengthened the formation of oligomers. Second, XRD and SEM results (Figures S2 and S3) demonstrate that when the LDO loading exceeded a certain amount, the excess LDO would destroy the ordered mesoporous structure of MCM-41 support, leading to the stacking of metal oxides. That would be another factor for the decreased selectivity of 70LDO.

## 3. Experiments

### 3.1. Catalyst Preparation

The LDO/MCM-41 composite (LDO loading = 50%) was prepared via an in situ method as follows: 2.7 mmol of cetyltrimethylammonium bromide was dissolved in 240 mL 15.0 mM NaOH solution at ambient temperature, followed by adding 30 mmol tetraethylorthosilicate (TEOS) under vigorous stirring for 1 h. Subsequently, 75 mL solution A containing 30 mmol Mg(NO_3_)_2_·6H_2_O and Al(NO_3_)_3_·9H_2_O (Mg/Al = 3, the molar ratio of nitrates to TEOS equaled to 1:1) was added dropwise into the above gel solution, and the pH of this solution was maintained to 9 by a solution B containing 30 mM NaOH. The resulting suspension was aged at 80 °C for 24 h, then filtered and thoroughly washed. Finally, the obtained LDH/MCM-41 precursor was dried and calcined at 550 °C for 4 h to obtain its oxide LDO/MCM-41. For comparison, naked LDO and MCM-41 was prepared using the same formula without adding silica source or nitrates. Moreover, LDO/MCM-41 with 30% and 70% loading (denoted 30LDO and 70LDO) were prepared by varying the molar ratio of nitrates, while other preparation conditions were kept constant.

### 3.2. Characterization

The X-ray diffraction (XRD) was recorded using a X-ray diffractometer (PANalytical X’Pert-Pro, Almelo, The Netherland) with a Cu Kα radiation (λ = 0.154 nm). The nitrogen adsorption–desorption isotherms were obtained using a Micromeritics ASAP 2010 apparatus (Micromeritics, Norcross, GA, USA) at 77 K. The specific surface area was calculated by using the Brunauer–Emmett–Teller (BET) equation. Pore diameter distribution was determined via the BJH model applied to the nitrogen adsorption isotherm. The morphologies of catalysts were characterized via scanning electron microscope (SEM) (Tescan Vega 3, Brno, Czech Republic). Temperature-programmed desorption of CO_2_/NH_3_ (CO_2_/NH_3_-TPD) was performed in Micromeritics AutoChem 2920 Automated Catalyst Characterization System (Micromeritics, United States). Typically, 0.1 g sample was first heated in He flow at 550 °C for 1 h and cooled to the adsorption temperature (80 °C for CO_2_, 100 °C for NH_3_). After the saturated adsorption, the sample was purged with He for 30 min to remove the physically adsorbed CO_2_/NH_3_. Then, the desorption began in He flow with a heating rate of 10 °C min^−1^. The amount of desorbed CO_2_/NH_3_ was detected with an OminiStar mass spectrometer (Pfeiffer, Asslar, Germany). Infrared spectroscopy with pyridine adsorption (Py-FTIR) for the sample was carried out on an Equinox 55 spectrometer (Bruker, Karlsruhe, Germany) equipped with a deuterated triglycine sulfate detector and an extended KBr beam splitter. The sample was evacuated in situ at 120 °C for 1 h. The spectrum was recorded at 120 °C.

### 3.3. Aldol Condensation

Typically, 0.2 g catalyst was mixed with 2.0 g CPO and placed in a hydrothermal reactor. The reactor was heated in an oil bath, which was preheated to a certain reaction temperature before stirring. After reaction, the liquid product was withdrawn and separated via centrifugation, then diluted with methanol. A gas chromatograph equipped with a HP-5 capillary column and a hydrogen flame-ionization detector was employed to analyze the liquid products.

## 4. Conclusions

In this work, hydrotalcite-derived MgAl mixed oxides supported on MCM-41 (LDO/MCM-41) was successfully synthesized via an in situ method and employed to catalyze the self-condensation of CPO for producing high-density fuel precursors. The hexagonal mesoporous structure and uniform pore size were well preserved in LDO/MCM-41. Compared with the bulk LDO, the LDO/MCM-41 composite had higher selectivity to target products (C10 and C15 oxygenates) even with less activity under the same reaction conditions. The yield of C15 was particularly enhanced on LDO/MCM-41, which was valuable for increasing the jet fuel density after hydrodeoxygenation. The catalyst stability was also improved due to the minimization of over-condensation. The outstanding performance of LDO/MCM-41 can be explained by the better dispersion of active sites and the more favorable acid-base properties. This work developed a new class of heterogeneous solid base catalysts with tunable physicochemical properties for aldol condensation.

## Figures and Tables

**Table 1:** Textural properties of LDO/MCM-41 and its control samples.

Catalyst	S_BET_ (m^2^ g^−1^)	Pore Volume (cm^3^ g^−1^)	Pore Size (nm)
MCM-41	738	0.65	3.5
LDO	150	0.18	15.2
LDO/MCM-41	541	0.50	6.1

**Table 2:** The properties of basic and acid sites on different samples.

Catalyst	Basic Sites (mmol g^−1^)				Total Acid Sites (mmol g^−1^)	B/L Ratio
	Weak	Medium	Strong	Total		
MCM-41	0.01	0	0	0.01	0.01	- ^a^
LDO	0.16	0.10	0.36	0.62	0.60	1.40
LDO/MCM-41	0.06	0.25	0.12	0.43	0.77	2.37

^a^ Null. Only Bronsted acid sites were detected on MCM-41.

**Table 3:** Catalytic activity of CPO condensation on LDO and LDO/MCM-41.

Catalyst	Conversion (%)	C10 Yield (%)	C15 Yield (%)	Carbon Balance (%)	TOF (h^−1^) ^c^
LDO (0.1 g, 2 h) ^a^	42.8	37.9	- ^d^	98	41
LDO (0.05 g, 4 h) ^b^	34.0	27.2	- ^d^	96	32
LDO/MCM-41(0.1 g, 2 h) ^a^	18.0	15.1	1.2	100	23
LDO/MCM-41(0.05 g, 4 h) ^b^	16.5	13.0	1.0	100	23

Reaction conditions: ^a^ 1.0 g CPO, 0.1 g catalyst, 160 °C, 2 h; ^b^ 1.0 g CPO, 0.05 g catalyst, 160 °C, 4 h; ^c^ Based on the total amount of basic sites; ^d^ not detected.

## Data Availability

Data are contained within the article and Supplementary Materials.

## Supplementary Materials

The following supporting information can be downloaded at: https://www.mdpi.com/article/10.3390/molecules28237920/s1, Figure S1: SEM-EDX mapping of LDO/MCM-41 (a) Si (b) O (c) Mg (d) Al (e) Merged image (f) SEM of mapped region; Figure S2: Low angle XRD patterns of LDO/MCM-41 composites with different LDO loading; Figure S3: SEM images of (a) 30LDO/MCM-41 and (b) 70LDO/MCM-41; Figure S4: Nuclear magnetic resonance spectroscopy of (a,b) C10 and (c,d) C15 oxygenates; Table S1: The acid-base properties and catalytic performance of LDO/MCM-41 composites with different LDO loadings.
