# Supplementary material for: Mesoporous-Layered Double Oxide/MCM-41 Composite with Enhanced Catalytic Performance for Cyclopentanone Aldol Condensation
Source: Molecules. 2023 Dec 3;28(23):7920. doi: 10.3390/molecules28237920 (PMC10708274; doi:10.3390/molecules28237920)
Supplement: Supplementary file 1 [file molecules-28-07920-s001.zip › molecules-2732730-supplementary.pdf]

Mesoporous layered double oxide/MCM-41 composite with enhanced catalytic performance for cyclopentanone aldol condensation

Jinfan Yang[a*], Ning Shang[a], Jiachen Wang[a], Huimin Liu[a]

[a] College of Bioresources Chemical and Materials Engineering, Shaanxi University of Science & Technology, Xi'an 710021, China

*Corresponding author: Dr. Jinfan Yang

College of Bioresources Chemical and Materials Engineering

Shaanxi University of Science & Technology

Xi'an, Shaanxi, 710021, China

Tel.: +86-029-86168575; Fax: + 86-029-86168575;

E-mail: yangjinfan@sust.edu.cn

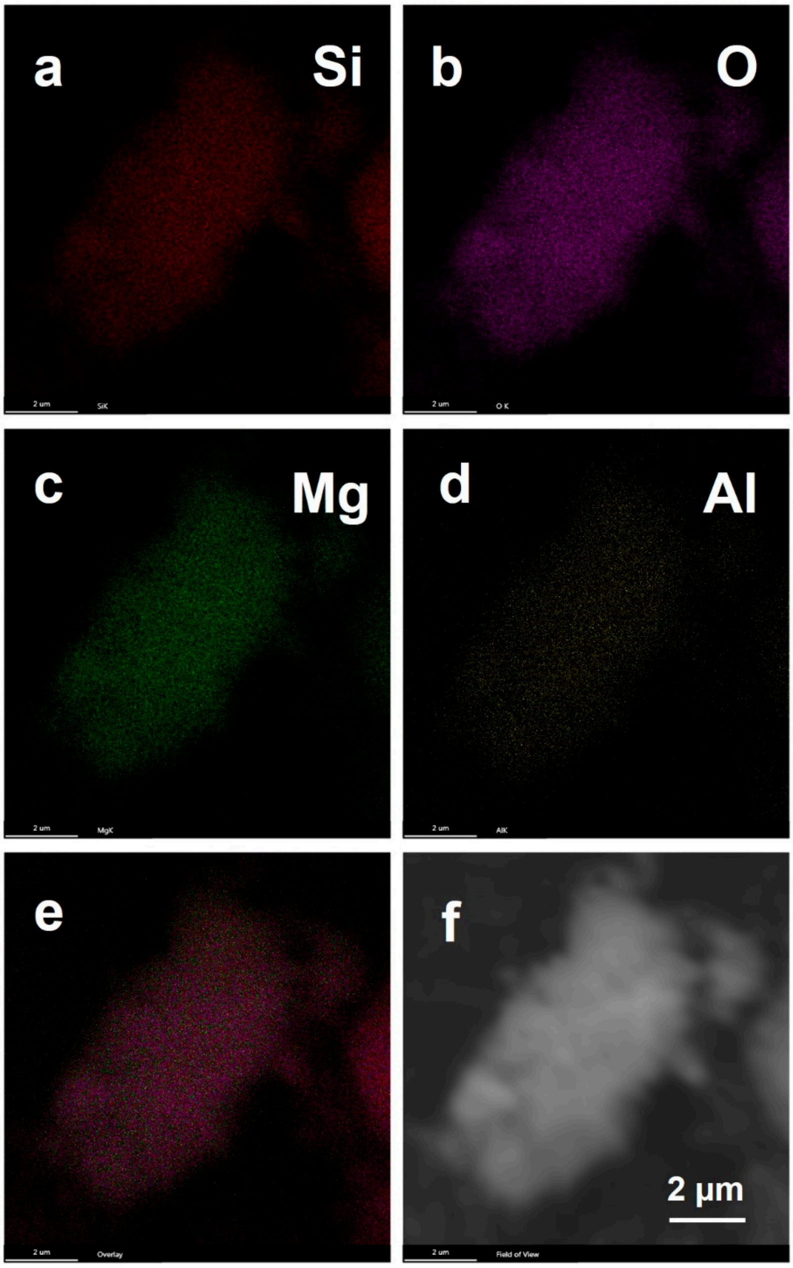

Figure S1. SEM-EDX mapping of LDO/MCM-41 (a) Si (b) O (c) Mg (d) Al (e) Merged image (f) SEM of mapped region.

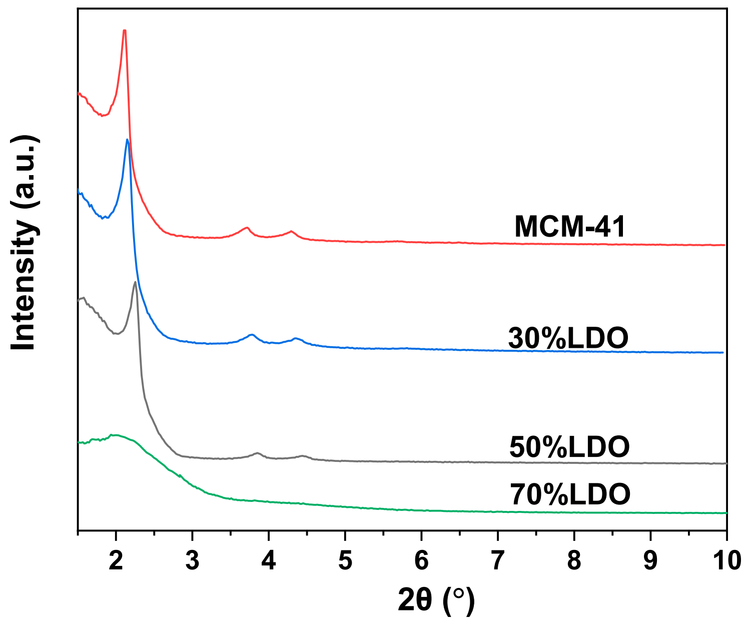

Figure S2 Low angle XRD patterns of LDO/MCM-41 composites with different LDO loading.

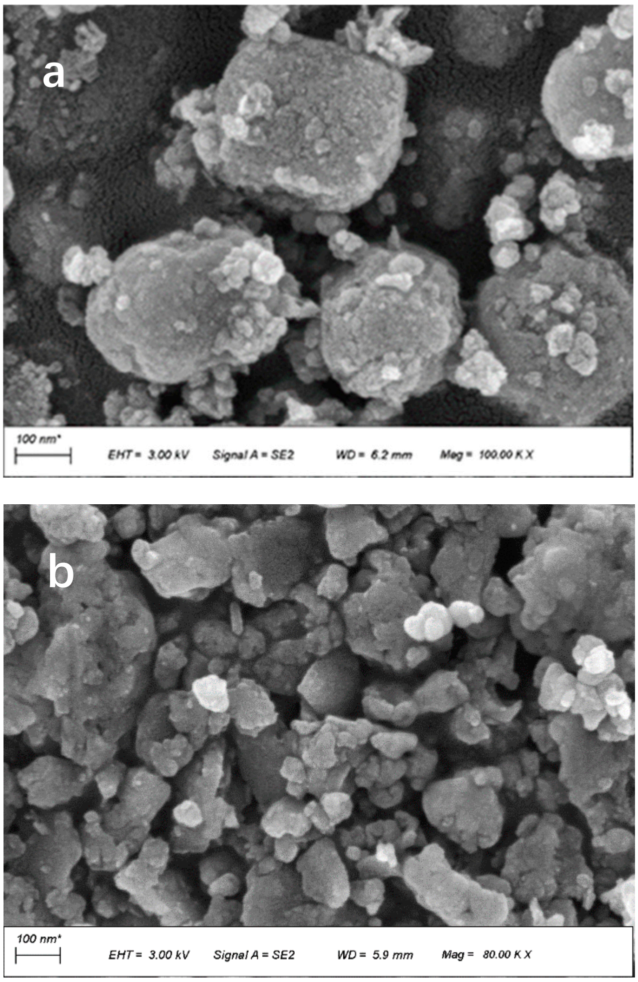

Figure S3 SEM images of (a) 30LDO/MCM-41, and (b) 70LDO/MCM-41.

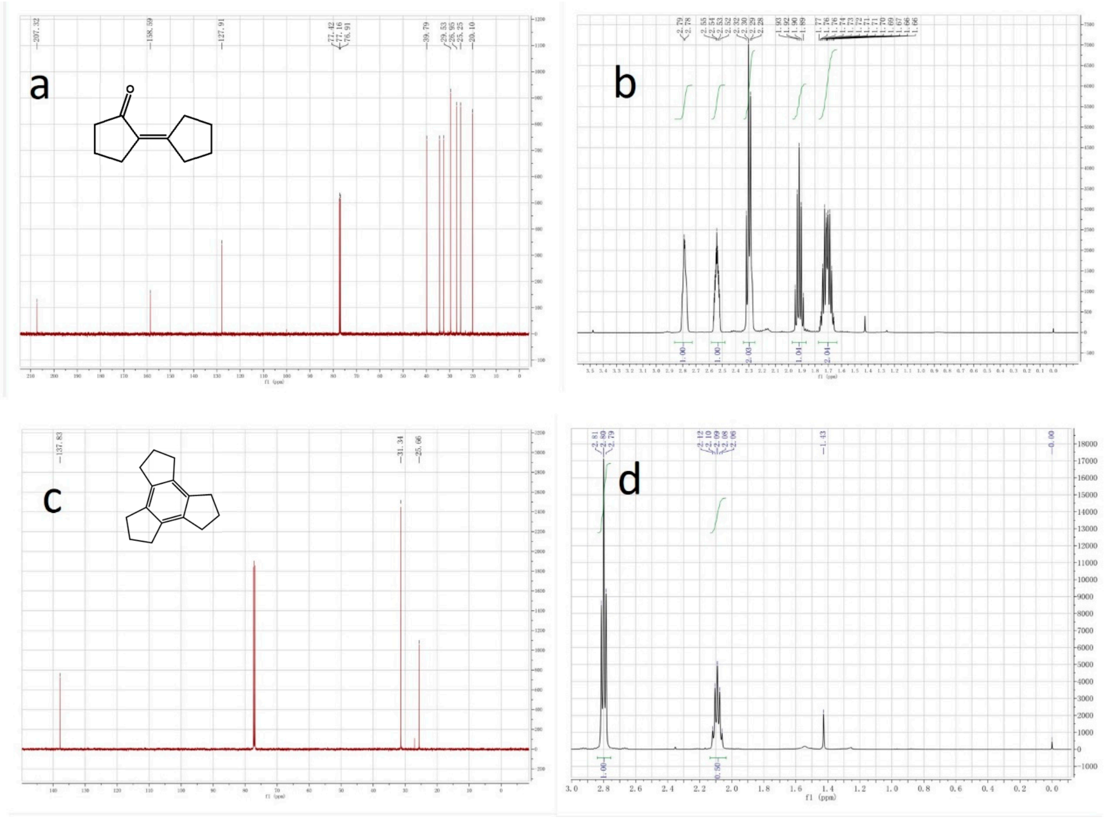

Figure S4. Nuclear magnetic resonance spectroscopy of (a-b) C10 and (c-d) C15 oxygenates.

Table S1 The acid-base properties and catalytic performance of LDO/MCM-41 composites with different LDO loadings.

LDO loading (%)	Base amount ($mmol\ g^{-1}$)				Conversion (%)	Yield (%)		Selectivity (%)
	Weak	Medium	Strong	Total		C10	C15	
30	0.04	0.11	0.05	0.20	56.3	48.6	6.1	97.1
50	0.06	0.25	0.12	0.43	77.8	60.0	15.2	96.6
70	0.13	0.16	0.27	0.56	89.0	65.5	13.0	88.2
